# Active juvenile systemic lupus erythematosus is associated with distinct NK cell transcriptional and phenotypic alterations

**DOI:** 10.1038/s41598-024-62325-3

**Published:** 2024-06-06

**Authors:** Anna Radziszewska, Hannah Peckham, Nina M. de Gruijter, Restuadi Restuadi, Wing Han Wu, Elizabeth C. Jury, Elizabeth C. Rosser, Coziana Ciurtin

**Affiliations:** 1 Centre for Adolescent Rheumatology Versus Arthritis at UCL UCLH and GOSH, London, UK; 2 Centre for Rheumatology Research, Division of Medicine, University College London, London, UK; 3 NHS North Thames Genomic Laboratory Hub, Great Ormond Street Hospital for Children, NHS Foundation Trust, London, UK

**Keywords:** Systemic lupus erythematosus, NK cells

## Abstract

While adaptive immune responses have been studied extensively in SLE (systemic lupus erythematosus), there is limited and contradictory evidence regarding the contribution of natural killer (NK) cells to disease pathogenesis. There is even less evidence about the role of NK cells in the more severe phenotype with juvenile-onset (J)SLE. In this study, analysis of the phenotype and function of NK cells in a large cohort of JSLE patients demonstrated that total NK cells, as well as perforin and granzyme A expressing NK cell populations, were significantly diminished in JSLE patients compared to age- and sex-matched healthy controls. The reduction in NK cell frequency was associated with increased disease activity, and transcriptomic analysis of NK populations from active and low disease activity JSLE patients versus healthy controls confirmed that disease activity was the main driver of differential NK cell gene expression. Pathway analysis of differentially expressed genes revealed an upregulation of interferon-α responses and a downregulation of exocytosis in active disease compared to healthy controls. Further gene set enrichment analysis also demonstrated an overrepresentation of the apoptosis pathway in active disease. This points to increased propensity for apoptosis as a potential factor contributing to NK cell deficiency in JSLE.

## Introduction

Systemic lupus erythematosus (SLE) is an autoimmune condition which causes significant morbidity and can be fatal when uncontrolled^1^. It is a complex, systemic disease, characterized by the production of autoantibodies against nuclear antigens and a strong type I interferon (IFN) signature^2^. There are numerous clinical manifestations which range from mild systemic, articular, and muco-cutaneous involvement to life-threatening complications such as renal nephritis or central nervous system involvement. Although the etiology of the disease is not completely understood, a combination of genetic predisposition and environmental factors are thought to be involved^1^. If SLE develops before the age of 18, it is classified as juvenile systemic lupus erythematosus (JSLE), which accounts for approximately 10–15% of all SLE cases^3^. JSLE is characterized by more aggressive disease with higher disease activity at onset, widespread organ involvement, and higher co-morbidity and mortality rates than in adult-onset SLE^4,5^. Due to lack of complete understanding of disease pathogenesis and limited therapeutic efficacy of available agents there is at present no cure for SLE. Current treatments rely on broad immunosuppression with corticosteroids and conventional disease modifying antirheumatic drugs, and in selected cases, B cell- and IFN-targeted therapies. Despite potential differences in pathophysiology and clinical presentation between adult and juvenile onset SLE, there are no age-tailored treatment approaches.

Natural killer (NK) cells are an essential part of the innate arm of the immune system that function to eliminate aberrant, virally infected or malignant cells^6^. In peripheral blood, NK cells are characterised by the absence of T cell receptor and CD3 on their surface and by expression of CD56^7^. CD56 expression marks the transition to the final mature NK cell phenotype and most immature NK cells transition first to a population with high levels of CD56 expression (CD56^bright^) before conversion to a low CD56 expressing (CD56^dim^) population. CD56^bright^ cells constitute approximately 5% of the total NK population with over 90% of NK cells expressing the CD56^dim^ phenotype. Downregulation of CD56 is associated with acquisition of cytotoxicity, while CD56^bright^ cells are potent producers of pro-inflammatory cytokines^7^.

NK cell activation is not antigen-recognition dependent and instead takes place via a series of interactions between multiple germ-line encoded inhibitory killer cell immunoglobulin-like **(**KIR) family receptors and activating receptors such as natural killer group 2D (NKg2D)^8^. NK cell activation through cell–cell interactions and cytokine exposure induces the expression of several surface molecules including CD25 and CD69^9^.

NK cells exert their effector functions of killing infected and tumorigenic targets through expression of cytotoxic mediators and cytokines. The main mechanism by which NK cells achieve cytolysis of adjacent cells involves release of perforin, granzymes and other cytotoxic enzymes from lytic granules. Killing can also occur by inducing death receptor-mediated apoptosis via the Fas and TRAIL pathways^7^.

Although direct cytotoxicity is their primary function, NK cells also secrete multiple cytokines, chemokines and growth factors including IFN-γ, tumour necrosis factor-α (TNF-α), CC-chemokine ligand 3 (CCL3), CCL4, CCL5, IL-8 and granulocyte–macrophage colony-stimulating factor (GM-CSF) to activate other immune cells and promote their migration to sites of inflammation^7^. NK cells are innate lymphoid cells (ILCs) and, along with ILC1, they belong to group 1 ILCs due to shared cytokine and transcription factor expression patterns^10^.

While many aspects of immune dysfunction have been studied extensively in JSLE, there is limited and at times contradictory evidence of how NK cells contribute to disease pathology. In children and adolescents with active JSLE, NK cells have been shown to have reduced absolute counts^11–13^ and frequencies^12,14,15^, which were associated with disease activity^12,14^ and renal involvement^14^, and to have diminished killing capacity^12^. In contrast, others found no association between NK frequencies and renal involvement or disease activity^15^. Similar conflicting findings have been reported in adult-onset SLE^16–21^, but it remains unclear if changes in the composition of the NK cell compartment are a consequence of the broader immune alterations associated with the disease and potentially treatment, or if they have a causal role in the disease pathogenesis.

In the present study, to address this, using flow cytometric and transcriptomic analysis, we have undertaken an in-depth exploration of NK cytotoxic capacity and function in peripheral blood in a large cohort of clinically well-characterised JSLE patients alongside age- and sex-matched healthy controls. We aimed to investigate the impact of JSLE activity on NK phenotype, cytotoxic capacity, and transcriptional profiles to provide novel evidence for the potential role of NK cells in JSLE pathogenesis.

## Results

### CD56^+^ NK cell frequencies and cytotoxic CD56^+^ NK populations are diminished in JSLE

**Figure 1:**
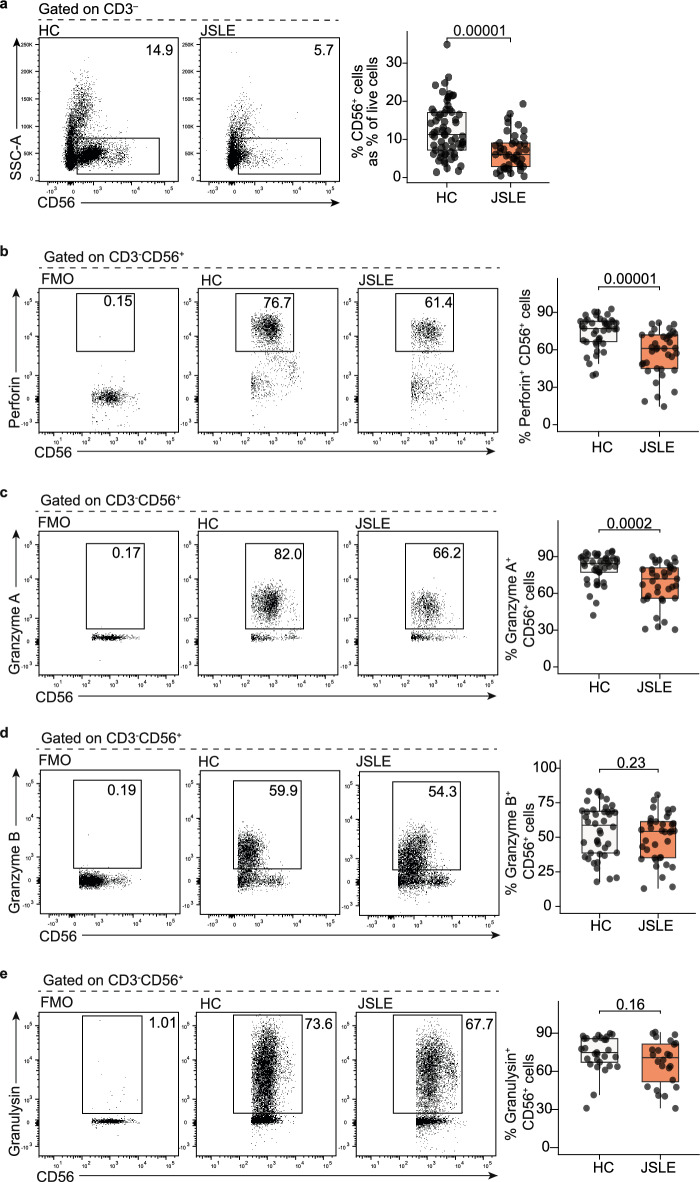
Reduced frequencies of CD56^+^ NK cells and perforin- and granzyme A-expressing NK cells in patients with JSLE. Representative flow plots and boxplots showing (**a**) total NK CD56^+^ cell frequencies in ex-vivo PBMCs expressed as percentage of live cells (HC n = 65, JSLE n = 42), percentage of CD56^+^ NK cells expressing (**b**) perforin, (**c**) granzyme A, (**d**) granzyme B and (**e**) granulysin in healthy controls (HC) and JSLE. Perforin, granzyme A, granzyme B: HC n = 42, JSLE n = 37, granulysin: HC n = 29, JSLE n = 24. Boxplots show median ± IQR. p-values calculated using Mann–Whitney U test or t-test as appropriate.

To examine the cytotoxic capacity of NK cells in patients with JSLE, frequencies of total CD3^-^CD56^+^ NK cells and NK cell populations expressing the cytotoxic molecules perforin, granzyme A, granzyme B, and granulysin were quantified in JSLE patients and healthy controls (see Table 1 for demographic and clinical features of patients and controls). In keeping with previous findings in JSLE and adult-onset SLE^17–20^, total CD56^+^ NK cells, expressed as percentage of live cells, were diminished in patients with JSLE (p = 0.00001, Fig. 1a). The frequencies of CD56^+^ NK cells expressing perforin (p = 0.00001, Fig. 1b), and granzyme A (p = 0.0002, Fig. 1c) were also significantly diminished in JSLE, while no differences were observed in the granzyme B- or granulysin-expressing cells (Fig. 1d,e). Further assessment of NK cell subsets found that CD56^dim^ NK cells, but not CD56^bright^ cells, were reduced in JSLE patients compared to controls (p = 0.00009, Supplementary Fig. S1). In keeping with their established role as a cytolytic subset, CD56^dim^ cells expressed more perforin and granzyme A than CD56^bright^ cells both in healthy subjects and in patients with JSLE, while lower levels of perforin (p = 0.00003) and granzyme A (p = 0.0004) expressing cells were observed in the CD56^dim^ subset in JSLE compared to healthy controls. These findings indicate that the reduction in perforin and granzyme A populations observed in total NK cells in JSLE may be due to a reduction in both the frequency of the CD56^dim^ NK subset and in perforin- and granzyme A-expressing CD56^dim^ NK cells.

**Table 1:** Demographic and clinical characteristics of healthy subjects and patients with JSLE.

	Healthy controls Number (%/range)	JSLE Number (%/range)	p-value
Total number	66	45	–
Female:male	45:21	33:12	0.67
Median age (years)	19.5 (15.2–32.2)	21.4 (15.4–29.8)	0.26
Ethnicity (%)			
White	32 (48%)	17 (38%)	0.33
Black	5 (8%)	7 (16%)	0.22
South Asian	12 (18%)	13 (29%)	0.25
East Asian	11 (17%)	3 (7%)	0.15
Other	6 (9%)	5 (11%)	0.75
Disease activity			
Median disease duration (years)	–	8.7	–
Median age at onset (years)	–	12.4	–
Global BILAG = 0	–	34 (76%)	–
Global BILAG = 1 (1 score C)	–	4 (9%)	–
Global BILAG = 8 (1 score B)	–	3 (7%)	–
Global BILAG = 9 (1 score B + 1 score C)	–	2 (4%)	–
Global BILAG = 18 (2 scores B + 1 score C)	–	1 (2%)	–
Global BILAG = 24 (3 scores B)	–	1 (2%)	–
Average SLEDAI	–	1.9 (0–10)	–
SLEDAI = 0	–	25 (56%)	–
SLEDAI = 2–4	–	14 (31%)	–
SLEDAI = 6–10	–	6 (13%)	–
Median serology (NR = normal range)			
Anti-dsDNA (IU/mL) (NR = < 50)	–	28.0	–
C3 (g/L) (NR = 0.9–1.8)	–	1.09	–
Lymphocyte count (10^9^/L) (NR = 1.2–3.5)	–	1.57	–
Treatment			
None	–	4 (9%)	–
Rituximab in the past year	–	0 (0%)	–
Rituximab ever	–	12 (27%)	–
Average duration since last rituximab treatment (years)	–	4.7	–
Hydroxychloroquine	–	38 (84%)	–
Methotrexate	–	5 (11%)	–
Azathioprine	–	8 (18%)	–
Mycophenolate Mofetil	–	19 (42%)	–
Prednisolone (any dose)	–	11 (24%)	–
Prednisolone ≥ 10 mg/day	–	6 (13%)	–
Cyclophosphamide in the past year	–	1 (2%)	–

An alternative gating approach defines NK cell subsets based on CD16 expression in addition to CD56 whereby the CD56^dim^ subset is defined as CD56^dim^CD16^+^ and the CD56^bright^ subset is CD56^bright^CD16^−^^22,23^. When defined in this way, frequencies of CD56^dim^CD16^+^ cells were also diminished in JSLE compared to healthy controls (p = 0.0024, Supplementary Fig. S2b) whilst no differences were observed in CD56^bright^CD16^−^ NK cells, or the frequencies of perforin- (Supplementary Fig. S2c) or granzyme A- (Supplementary Fig. S2d) expressing cells in either NK subpopulation.

### NK cell cytotoxicity declines with age in healthy individuals but not in patients with JSLE

**Figure 2:**
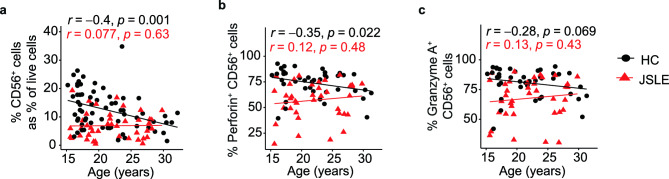
CD56^+^ NK cells and perforin^+^ and granzyme A^+^ CD56^+^ cells decline with age in healthy subjects. Scatter plots showing correlations between age at time of sampling and frequencies of (**a**) CD56^+^ NK cells expressed as percentage of live cells (HC n = 65, JSLE n = 42), NK cells expressing (**b**) perforin and (**c**) granzyme A (HC n = 42, JSLE n = 37) in healthy controls and JSLE. Spearman’s rho correlation coefficients and the associated p-values are shown.

As factors such as age or sex are known to have an impact on the immune system^24,25^, we assessed whether there were any associations between NK populations and these parameters in JSLE patients vs healthy controls. Notably, whilst the frequency of CD56^+^ NK cells as well as perforin^+^ and granzyme A^+^ NK cells were found to decrease with age in healthy controls (Fig. 2a–c), the correlation between NK population frequency and age was lost in patients with JSLE. This may indicate that disease overrides the age-related physiological processes regulating NK population expansion, suggesting a general dysregulation in NK homeostatic mechanisms in JSLE patients. Notably, stratification of JSLE patients and controls by sex revealed no significant differences in total NK cells, perforin-, or granzyme A- expressing CD56^+^ cells (Supplementary Fig. S3).

### NK cell function is altered in JSLE patients compared to healthy controls

**Figure 3:**
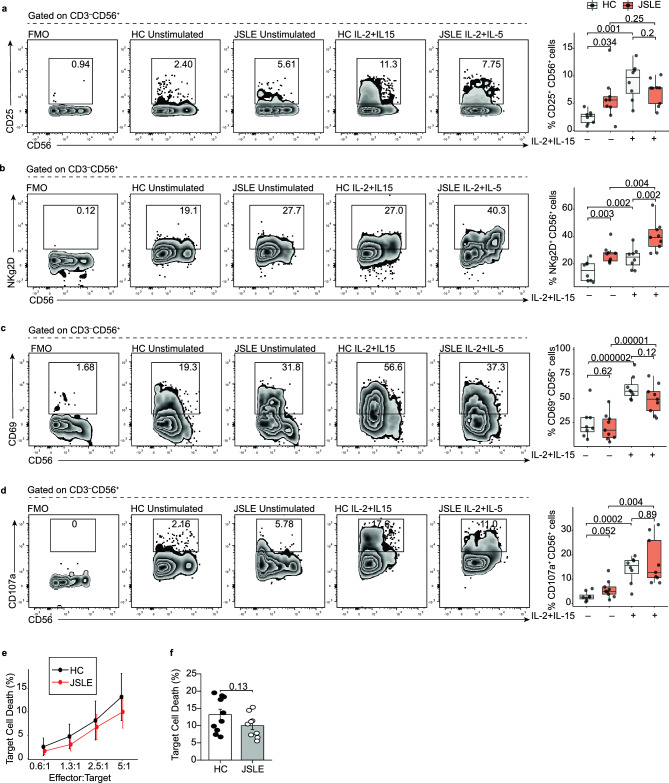
Increased frequencies of NK cells expressing activation markers in JSLE. Boxplots showing frequencies of (**a**) CD25^+^ (**b**) NKg2D^+^ (**c**) CD69^+^ (**d**) CD107a^+^ NK cells (HC n = 8, JSLE n = 9), with and without IL-2 and IL-15 stimulation. Box plots shown median ± IQR. p-values calculated using paired or unpaired t-tests or Mann–Whitney U tests. (**e**) Cytotoxicity of NK cells from healthy controls and JSLE patients against K562 cells at increasing effector:target (E:T) ratios measured in a 4-h flow cytometry assay. (**f**) Bar plot quantifying NK cytotoxicity against K562 cells at 5:1 E:T ratio. p-value calculated using two-sided Student’s t-test.

To investigate whether the reductions in CD56^+^ NK and cytotoxic NK populations were accompanied by changes in NK cell function in JSLE, we next assessed the frequency of NK cells expressing activation markers CD25, CD69, activation receptor NKg2D and degranulation marker CD107^26^ in JSLE patients compared to healthy controls both ex vivo (unstimulated) and following stimulation with prototypical NK activating factors IL-2 and IL-15. In unstimulated cells, frequencies of CD56^+^ NK cells expressing CD25 and the activation receptor NKg2D were significantly increased in JSLE (Fig. 3a,b, p = 0.034, p = 0.003, respectively) while no differences were detected in CD69^+^ NK cell frequencies compared to controls (Fig. 3c). In keeping with the reduction in cytotoxic molecules, in JSLE patients there was an increase in the proportion of NK cells expressing the degranulation marker CD107a (Fig. 3d).

Upon stimulation with IL-2 and IL-15, the expression of all NK activation and degranulation markers increased both in healthy controls and in JSLE (Fig. 3a–d), with the notable exception of CD25 which was already high in unstimulated JSLE (Fig. 3a). Frequencies of NKg2D expressing NK cells (Fig. 3b) remained significantly higher in JSLE compared to healthy controls after stimulation with cytokines (p = 0.002). These observations suggest that NK cells in JSLE may be in a heightened state of activation accompanied by increased degranulation and reduction in cytotoxic molecules.

Finally, to further examine the functional status of CD56^+^ NK cells in JSLE patients and healthy controls, NK cytotoxic activity was assessed in a flow cytometric assay against HLA Class I-negative K562 target cells. NK cells from patients with JSLE tended to exhibit reduced capacity to lyse K562 targets at all effector:target (E:T) ratios tested compared to NK cells from healthy controls (Fig. 3e,f). However, the differences were not statistically significant, possibly due to high variability in target lysis within both the control and the JSLE group.

### Reduction in cytotoxic CD56^+^ NK cell frequency is driven by JSLE activity

**Figure 4:**
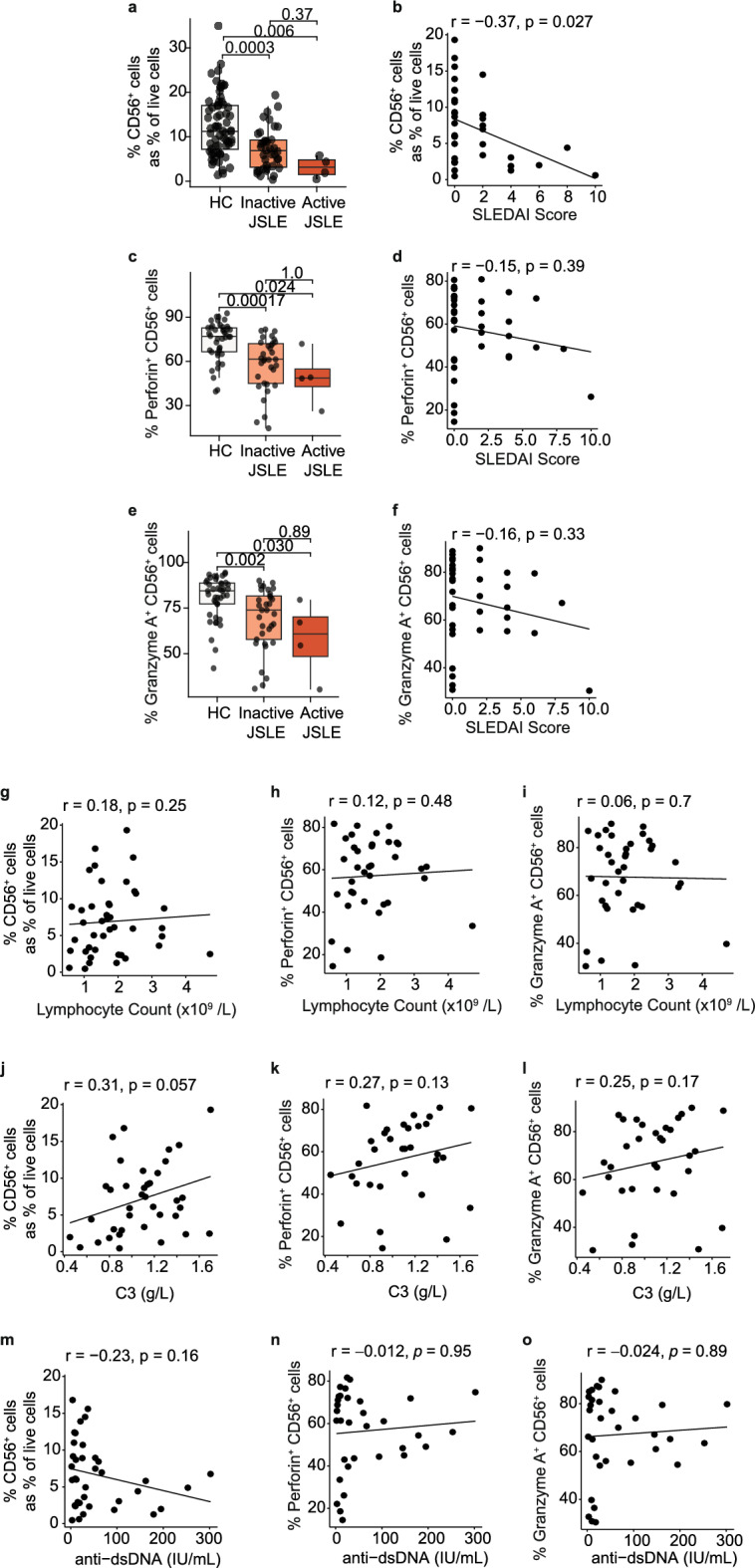
Frequency of CD56^+^ NK cells correlates with JSLE disease activity (SLEDAI). Boxplots of frequencies of (**a**) total NK cells expressed as percentage of all live cells (HC n = 65, Inactive JSLE n = 38, Active JSLE n = 4), (**c**) perforin^+^ NK cells, and (**e**) granzyme A^+^ NK cells (HC n = 42, Inactive JSLE n = 33, Active JSLE n = 4) stratified based on SLEDAI score. Active JSLE = SLEDAI > 4, Inactive JSLE = SLEDAI ≤ 4. Boxplots show median ± IQR. p-values calculated using Dunn’s test with Bonferroni correction. Correlations between SLEDAI scores and (**b**) total NK cell expressed as percentage of live cells (n = 36), (**d**) perforin^+^ (n = 32), and (**f**) granzyme A^+^ NK cell frequencies (n = 32). Scatter plots showing correlations in JSLE between lymphocyte counts and (**g**) CD56^+^ NK cell frequencies expressed as percentage of live cells (n = 42) and NK cells expressing (**h**) perforin (n = 37) and (**i**) granzyme A (n = 37), C3 levels and (**j**) CD56^+^ NK cells as percentage of live (n = 38) and NK cells expressing (**k**) perforin (n = 33) and (**l**) granzyme A^+^ (n = 33), dsDNA antibody levels and (**m**) NK cells as percentage of live (n = 37) and NK cells expressing (**n**) perforin (n = 33) and (**o**) granzyme A (n = 33). Spearman’s rho correlation coefficients and their associated p-values are shown.

JSLE is a highly heterogeneous disease and parameters such as clinical and serological disease activity, organ involvement, and medication use may influence the immune phenotype. To determine the impact of JSLE disease activity on NK cell immunophenotypes found to be differentially expressed in JSLE, frequencies of total, perforin^+^ and granzyme A^+^ NK cells were compared in healthy controls and JSLE patients stratified based on their SLEDAI-2K (systemic lupus erythematosus disease activity index-2000) scores (see “Methods”, Table 1)^27^. Patients with SLEDAI > 4 were defined as having active disease as per validated definitions^28^. CD56^+^ NK cell frequencies were diminished in JSLE patients with low disease activity compared to controls (p = 0.0003) and reduced further still in patients with active disease (p = 0.006, Fig. 4a). A negative correlation was observed between CD56^+^ NK cell frequencies and SLEDAI scores (Fig. 4b). Similar trends were observed in the cytotoxic perforin^+^ and granzyme A^+^ CD56^+^ cell populations (Fig. 4c–f), although statistical significance was not achieved. No statistically significant differences were found in NK cell populations in patients stratified based on their global BILAG (British Isles Lupus Assessment Group) index score^29^ or across any of the organ domains comprising the BILAG index (Supplementary Fig. S4).

In addition to disease scores, lymphocyte counts and serological disease activity markers such as complement 3 (C3) and anti-double stranded DNA antibodies (anti-dsDNA) are routinely measured in clinical practice to assist patient management with rising anti-dsDNA levels and falling C3 levels potentially indicative of a disease flare^30^. No statistically significant correlations were observed between NK cell populations and lymphocyte counts (Fig. 4g–i), C3 levels (Fig. 4j–l) or anti-dsDNA levels (Fig. 4m–o), suggesting that the differences in NK cell populations observed between healthy controls and JSLE patients stratified by disease activity could be driven by clinical activity in patients with SLEDAI > 4 compared to patients with serological activity alone or in complete remission (SLEDAI ≤ 4).

Common treatments for SLE such as mycophenolate mofetil (MMF), azathioprine (AZA), hydroxychloroquine (HCQ), and prednisone, have been shown to down-modulate NK cell function, cytotoxicity, and proliferation^31–34^. To investigate if the reductions in NK cell populations seen in JSLE, and specifically those associated with a higher SLEDAI score, were confounded by treatment, patients were dichotomised based on their exposure to various JSLE medications at the time of sampling and compared to healthy controls. No significant differences were found between the frequencies of CD56^+^ total, perforin^+^, and granzyme A^+^ NK cell populations in patients treated or not with MMF, HCQ, or prednisolone (Supplementary Fig. S5). In keeping with published results in adult-onset SLE^34^, AZA treatment was associated with a reduction of total NK cells in JSLE (p = 0.019, Supplementary Fig. S5d). However, AZA treatment did not appear to influence cytotoxic NK populations (Supplementary Fig. S5e,f). Taken together, these findings suggest that although reductions in CD56^+^ NK cells may be associated with disease activity as well as AZA treatment, the reduction in frequencies of NK cell cytotoxic populations expressing perforin and granzyme A in JSLE was not due to treatment, but rather, was a feature of the disease.

### Transcriptional analysis of CD56^+^ NK cells reveals upregulation of interferon-α inducible genes and downregulation of exocytosis pathways in patients with active JSLE

To elucidate the possible mechanisms underpinning diminished NK cell cytotoxicity in JSLE and specifically those associated with SLEDAI disease activity, transcriptomic analysis was performed using sorted CD56^+^ NK cells from 6 healthy controls, 7 JSLE patients with low disease activity (SLEDAI score ≤ 4), and 5 JSLE patients with more active disease (SLEDAI score > 4) (see Supplementary Table S1 for cohort clinical details). The 3 subject groups were very well matched for sex, age and ethnicity (Supplementary Table S2). With the exception of 2 active JSLE patients, all individuals included in the transcriptional analysis cohort were also included in the NK cell immunophenotyping cohort. The cohorts were comparable in terms of demographic and clinical characteristics with no statistically significant differences in sex, age, disease activity or AZA treatment across the two cohorts (Supplementary Table S3).

**Figure 5:**
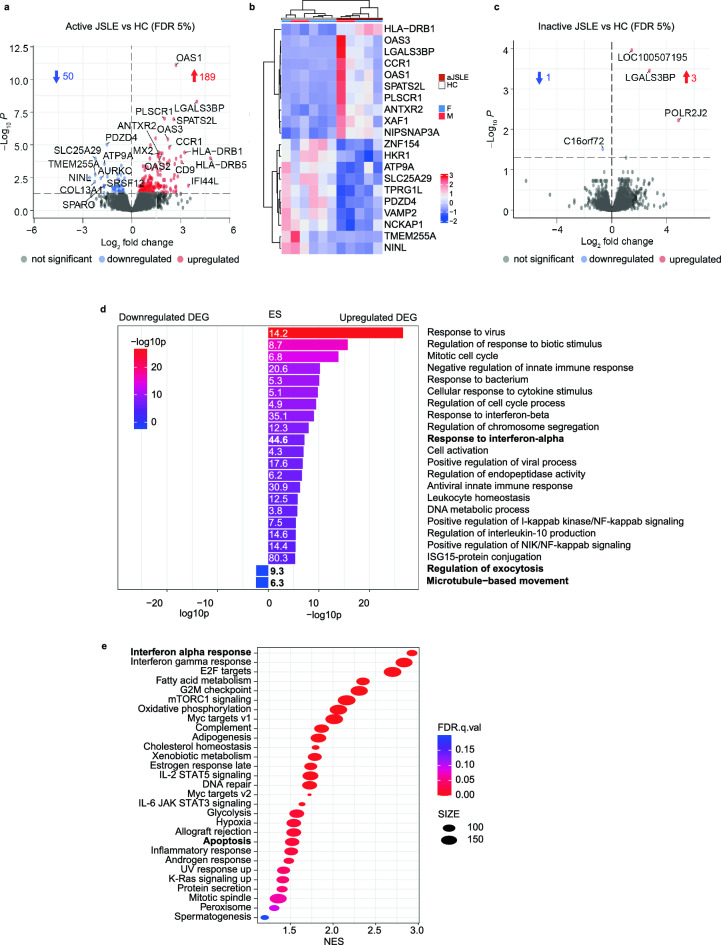
Transcriptional differences in NK cells are more pronounced in active JSLE. Volcano plots showing differences in gene expression from RNA sequencing in NK cells from (**a**) active JSLE (n = 5) vs healthy controls (n = 6) and (**c**) inactive JSLE (n = 7) vs healthy controls (n = 6). Blue and red points represent statistically significant DEGs below the FDR adjusted p-value threshold of 0.05. Blue and red arrows indicate number of statistically significant downregulated and upregulated genes, respectively. (**b**) Hierarchical clustering heatmap (Pearson’s) of normalised gene counts of top 10 upregulated and downregulated (p.adj < 0.05) DEGs between aJSLE (active JSLE) vs healthy controls (M = male, F = female) (**d**) Pathway analysis plot showing -log10p values and enrichment scores (ES) of top 20 enriched pathway ontology terms in NK cells between active JSLE and healthy controls using the 189 significantly upregulated and 50 significantly downregulated genes. (**e**) Dot plot of GSEA displaying FDR q.value (p-value normalised for gene set size and multiple testing) and normalized enrichment scores (NES) of pathway ontology terms in NK cells of active JSLE vs healthy controls using the entire gene list. Dot size indicates number of genes in the gene set.

Analysis of coding transcripts from RNA sequencing (RNA-Seq) of NK cells from patients with active JSLE compared with healthy controls revealed that 189 transcripts were upregulated in active JSLE (FDR adjusted p < 0.05) while 50 transcripts were downregulated (FDR adjusted p < 0.05) (Fig. 5a). Top 10 upregulated and downregulated genes from this analysis (Fig. 5b) along with their functions are shown in Supplementary Table S4. A similar analysis comparing transcripts from patients with low disease activity with transcripts from healthy controls showed that only 3 genes were upregulated and 1 gene was downregulated in JSLE (FDR adjusted p < 0.05) (Fig. 5c, Supplementary Table S5). Analysis of transcripts from patients with active disease compared to those with low disease activity revealed 18 differentially expressed genes between the two groups (Supplementary Fig. S6, Supplementary Table S6). Thus, just as differences in NK cell cytotoxic phenotypes were more pronounced in patients with active disease, differences in the NK cell transcriptome were also much more evident in active JSLE than in low disease activity.

Pathway enrichment analysis of differentially expressed genes (DEGs) in active JSLE vs healthy controls revealed statistically significant GO BP ontology terms related to upregulation of interferon-α (IFN-α) responses (p = 7.1 × 10^–8^) and downregulation of exocytosis (p = 0.004) and microtubule-based movement (p = 0.004) (Fig. 5d). These findings were in keeping with other published reports as type 1 IFN Gene Signature (IGS) is a well-reported feature of multiple immune cell populations in SLE and JSLE^35^, while downregulation of genes involved in exocytosis and microtubule-based movement may point to disruptions in NK cell cytotoxic granule exocytosis in JSLE. Pathway enrichment analysis results were confirmed using gene set enrichment analysis (GSEA), which takes the entire transcript list into account (Fig. 5e). As expected, IFN-α response was again the most prominent pathway over-represented in active JSLE and GSEA additionally identified changes in metabolic pathways (fatty acid oxidation, oxidative phosphorylation, glycolysis and cholesterol homeostasis) suggesting that metabolic dysfunction may be occurring in NK cells in active JSLE. Genes involved in apoptosis were also overrepresented in GSEA pointing to increased apoptosis as a potential cause of reduction in NK cell frequencies in the disease.

### Reduction in NK cell populations in JSLE may be due to increased apoptosis

**Figure 6:**
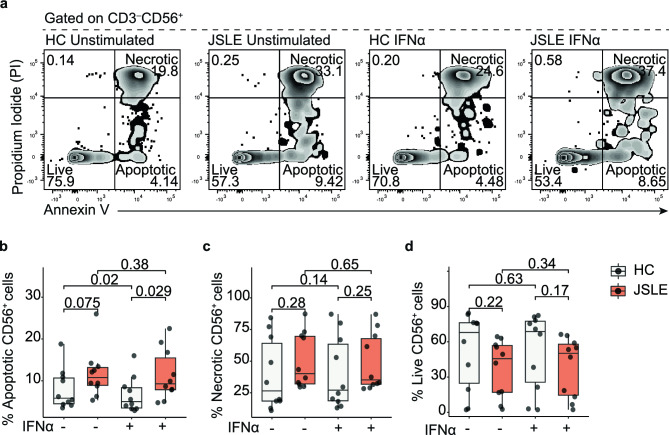
NK cells in JSLE are more susceptible to apoptosis in the presence of IFN-α. (**a**) Representative flow plots and boxplots showing Annexin V and PI staining. Boxplots showing frequencies of (**b**) apoptotic, (**c**) necrotic, and (**d**) live CD56^+^ NK cells in healthy controls (n = 10) and JSLE patients (n = 10) with and without stimulation with IFN-α for 48 h. Box plots shown median ± IQR. p-values calculated using paired or unpaired t-tests or Mann–Whitney U tests.

Finally, to explore the possibility that increased cell death may be one of the underlying causes of the reduction of NK cell frequencies in JSLE, we next assessed NK cell apoptosis using flow cytometric staining with Annexin V/propidium iodide (PI) (Fig. 6a). Although it did not meet the threshold of statistical significance, the frequency of apoptotic NK cells tended to be higher in JSLE compared to healthy controls (Fig. 6b) while the frequencies of necrotic (Fig. 6c) and live (Fig. 6d) cells were unchanged. Unfortunately, due to prevalent lymphopenia and the profound deficiency in NK cells observed in more active patients, we were only able to perform these assays in patients with low disease activity. However, when we assessed the difference in the frequency of apoptotic cells in JSLE patients compared to healthy controls following exposure to IFN-α, there was an increased frequency of apoptotic cells in JSLE compared to healthy controls (Fig. 6b). Collectively these findings suggest that increased propensity for apoptosis may, at least in part, explain the reduction of CD56^+^ NK cells in JSLE and that this is exacerbated by exposure to high type 1 IFN which has been previously associated with heightened disease activity^35^.

## Discussion

The role of NK cells in JSLE and adult-onset SLE has not been well characterised and studies in human lupus are mainly descriptive and at times contradictory. Although absolute numbers and frequencies of NK cells have been consistently reported to be diminished in JSLE and adult-onset SLE^11–15^, some have found associations between their numerical deficiency and disease activity or renal involvement^12,14^ while others have found no evidence for such associations^15^. In this study, to address this heterogeneity and investigate the NK cell immunophenotype in a large cohort of well-characterised JSLE patients, we assessed the levels of multiple cytotoxic markers in JSLE patients versus age- and sex-matched controls. We have found that frequencies of NK cells expressing perforin and granzyme A were reduced in JSLE patients along with reduction in total NK cell frequencies, as well as diminished frequencies of the mature cytotoxic CD56^dim^ NK subset. The major driver of these phenotypic differences was disease activity, which was validated at the transcriptional level.

Some of the discrepancies between our findings and published literature both in JSLE and adult-onset SLE, may be explained by the inherent disease heterogeneity as well as more specific genetic, environmental, and socio-economic factors which are unique to every cohort. In addition, methodological considerations such as gating strategies, reagents, and differences between assays used to quantify cytotoxic function could contribute to result variability.

Our study validated findings reported in the literature related to reductions in total NK cell frequencies^12,14^ in patients with JSLE and confirmed that the association between reduced NK cell frequencies and azathioprine treatment seen in adult-onset SLE^34^ is also present in our JSLE cohort. Furthermore, we found that the reductions in NK cell populations correlated with increased JSLE disease activity, despite exploring a JSLE patient cohort with predominantly well-controlled disease (only 4 patients had SLEDAI scores above 4), which is potentially relevant in supporting the role of NK cells in disease pathogenesis.

A previous study found an increase in CD56^bright^ NK cell frequency in JSLE which correlated positively with clinical and serological disease activity^14^, but we found no evidence of this in our cohort. The reasons for this discrepancy are unclear, but may be due to differences in methodology or cohort characteristics.

Possible explanations for the reduction in NK cytotoxic populations include increased migration of CD56^dim^ NK cells into tissues. In support of this hypothesis, NK cells have been found in kidney infiltrates in murine lupus models^36^, and transcriptomic analysis of kidney tissue has identified the presence of CD56^dim^ and CD56^bright^ clusters in patients with lupus nephritis^37^. In addition, peripheral CXCR3^+^CD56^dim^ NK cells are reportedly reduced in active SLE patients. As CXCR3 is involved in NK cell trafficking, the reduction in these cells could reflect their shift from the periphery to target organs where they may promote tissue damage^21^. A correlation between lupus nephritis and reduction in circulating NK cells has been reported both in JSLE and adult-onset SLE^14,17^. However, our observation that in JSLE the CD56^dim^ cells that remain in peripheral blood have diminished expression of perforin and granzyme A, implies that in addition to NK cell quantitative insufficiency, possibly due to migration of CD56^dim^ cells into target tissues, there are other mechanisms that contribute to NK cell deficiency in JSLE.

Our RNA-Seq transcriptomic analysis in NK cells from active JSLE patients compared to healthy controls identified differences in expression in genes involved in apoptotic pathways. In line with these findings, we observed that NK cells from JSLE patients had an increased propensity toward apoptosis in the presence of IFN-α. This, combined with findings in adult-onset SLE which suggest that plasma from patients with active disease can induce NK cell apoptosis in an IFN-α dependent manner^36^, implies that NK cells are more susceptible to apoptosis in the context of active disease and that apoptosis may at least in part contribute to the numerical deficiency of NK cells observed in JSLE. Transcriptomic analysis also identified metabolic dysfunction as a potential mechanism contributing to NK functional alterations in active JSLE. In support of this, intracellular oxidative stress has been reported to be increased in NK cells and to correlate positively with SLEDAI disease activity in adult-onset SLE^38^. CD56^dim^ cell frequencies were additionally found to correlate positively with expression of Nrf-2, a protein involved in protection from oxidative stress^38^. Mitochondrial reactive oxygen species (ROS) production as well as endogenous apoptosis have also been shown to be increased in some subsets of CD56^dim^ cells in adult-onset SLE^39^. These studies, however, have all focused on adult-onset SLE and NK metabolism in JSLE remains largely unexplored and could be an interesting focus for future studies.

An additional important finding from our study included the observation that NK cell cytotoxic populations declined with age in healthy controls while in JSLE, the reduction in NK cytotoxic phenotype associated with disease seems to override any differences associated with age. This underscores the importance of age-matching patient and control cohorts, especially in studies involving children and young adults. It also highlights the plasticity of the developing immune system and may suggest that mechanisms triggering and driving disease progression can change along with the development and aging of the immune system. Finally, this observation could point to key differences between adult-onset SLE and JSLE and therefore discrepancies amongst studies. While the lack of comparison with adult-onset SLE data precludes the possibility of reaching definitive conclusions, given the reduction in NK cytotoxicity with age in healthy controls, it is possible that the differences in cytotoxic NK cell populations observed in JSLE do not exist in adult-onset SLE.

Although our study focused on NK cell cytotoxicity, NK cells also secrete immunomodulatory cytokines, notably IFN-γ and TNF-α. IFN-γ inhibits viral replication, boosts macrophage function, and increases major histocompatibility complex expression to enhance recognition of infected cells by T cells^40^. TNF-α induces apoptosis of infected and malignant cells through interaction with TNFR-1 (TNF receptor 1) and promotes the immune response through TNFR-2-mediated proliferation signals in T cells and B cells^41^. NK cells in active patients with adult-onset SLE have been shown to express high amounts of IFN-γ^42^. Intracellular production of IFN-γ by NK cells was directly correlated with serum levels of IFN-α, which, along with TNF-α, has been shown to induce IFN-γ synthesis^43^. In contrast, the percentage of TNF-α-expressing NK cells reportedly reduced in active patients with adult-onset SLE^44^. The cytokine expression profile of NK cells in JSLE remains to be investigated.

In summary, our study has shown for the first time that NK cell cytotoxic capacity is diminished in JSLE and confirmed previous reports that NK cells frequencies are reduced in JSLE, in particular in active disease. Transcriptomic analysis of NK cells from active JSLE patients compared to healthy controls established that disease activity was the main driver of differential gene expression in JSLE and identified potential new avenues of research to explore the underlying factors behind NK cell defects in JSLE. Strategies aimed at boosting NK cell cytotoxicity have already been proposed in SLE^45^ and elucidation of precise mechanisms of NK cell dysfunction in JSLE will allow us to develop new targeted therapies in the future.

### Limitations of study

Notable limitations of our study are focused on constrained opportunities to obtain numerous samples from children and adolescents with active disease. As such, the majority of our cohort had well-controlled disease. In addition, in JSLE in general and particularly in active JSLE, lymphopenia and reduction in NK cells make detailed functional experiments, especially those requiring purified NK cells, inherently difficult. Thus, despite the pronounced reductions in cytotoxic NK cells observed in JSLE, we only observed a modest reduction in NK cell cytotoxic function, possibly due to the fact that functional assays were performed on patients with inactive disease. Furthermore, our findings represent the NK phenotype and function in peripheral blood only; analysis of tissues from organs affected by the disease is required to ascertain if NK cells contribute to disease pathology at those disease sites.

## Methods

### Patient and control samples

All research participants were recruited with informed age-appropriate consent as approved by the London-Harrow Research Ethics Committee (study reference: 11/LO/0330) and research was conducted in accordance with the Declaration of Helsinki and NHS HRA (National Health Service Health Research Authority) guidelines. Patients diagnosed with JSLE were recruited from the adolescent rheumatology clinics at University College London Hospital (UCLH). Healthy controls aged 15–16 years old were recruited from pre-assessment dental and urological surgery clinics at UCLH. Blood from these participants was taken under general anesthetic at the time of surgery. Healthy control samples from young people aged 16 and over were obtained from volunteers from the community. Healthy controls were excluded if they had viral symptoms or received any vaccine in the previous 3 weeks. A total of 20 mL of blood was taken from each participant. Whole blood destined for PBMC isolation was collected in tubes coated with sodium heparin. 45 JSLE patients and 66 age matched healthy controls were included in the study. Clinical and demographic data for all participants is shown in Table 1.

### PBMC isolation from blood

#### PBMC isolation

Peripheral blood mononuclear cells (PBMCs) were isolated by Ficoll gradient centrifugation using SepMate™ (StemCell) tubes, as described^46^. Viable cells were counted by trypan blue exclusion and cryopreserved in 10% DMSO, 90% FBS freezing media for long term storage in vapour phase liquid nitrogen.

### Flow cytometry

PBMC populations were phenotyped by multi-parameter flow cytometry using commercially available fluorochrome conjugated antibodies. PBMCs were thawed in complete media consisting of RPMI-1640, 10% FBS, penicillin (100 IU/ml) and streptomycin (100 μg/ml) and plated in 96-well plates at a density of 0.5 × 10^6^/well. The average post-thaw cell viability was 73%. All subsequent steps were performed at room temperature (RT) with incubations performed in the dark. During wash steps, the plates were centrifuged at 500 g for 5 min.

#### Surface staining

To exclude dead cells, PBMCs were stained with UV Live/Dead fixable blue stain (Thermo Fischer Scientific) or Ghost Dye Violet 510 (Tonbo Biosciences) for 15 min. This was followed by a wash in FACS buffer (phosphate buffered saline (PBS) + 1% FBS + 2 mM EDTA). Surface staining was performed by incubating with PE-Cy7-CD56 and BUV805-CD3 antibodies in FACS buffer for 20 min. After a wash step, if intracellular staining was not required, cells were fixed by incubation for 15 min in 2% paraformaldehyde and washed twice in FACS buffer.

#### Intracellular staining

For intracellular staining of cytotoxic molecules, cells were incubated for 20 min in fixation buffer (ebioscience^TM^Foxp3 /Transcription Factor Staining Buffer Set, Thermo Fisher, 00-5523-00). Cells were then washed in permeabilization buffer and incubated for 40 min with intracellular antibodies PE-Granulysin, FITC-Granzyme A, PerCP-Cy5.5-Perforin, AF700-Granzyme B in permeabilization buffer. This was followed by a wash in permeabilization buffer and a final wash in FACS buffer.

#### Data acquisition and analysis

Data was acquired using an LSR II flow cytometer running FACSDiva software. As many events as possible were acquired. Data was analysed using FlowJo software (TreeStar). Population frequencies were expressed as percentage of parent population in all analyses, unless indicated otherwise (notably, total CD56^+^ NK cell frequencies which were expressed as percentage of all live cells).

### Primary cell culture assays

#### NK cell activation assay

As described by Veluchamy et al.^47^, PBMCs from healthy controls and patients with JSLE were incubated overnight in complete media with and without IL-2 (1000 IU/ml) and IL-15 (10 ng/ml). PE-CD107a antibody was also added to all wells to detect NK cell degranulation. Brefeldin (5 µg/mL) and monensin (5ug/mL) were added 4 h prior to the end of the incubation to inhibit protein secretion. At the end of the incubation period cells were stained with UV Live/Dead fixable blue stain, followed by surface staining with APC-Cy7-CD3, APC-CD25, PE-Cy7-CD56, FITC-CD14, BV785-CD69, BUV737-CD16, PEDazzle-NKg2D antibodies as described.

### K562 killing assay

NK cell cytotoxic function was assessed using a flow cytometric assay as described by Kandarian et al^48^. The assay involves incubating NK cells with fluorescently labelled tumour targets (K562) known to be sensitive to NK cell cytotoxicity and quantifying the percentage of lysed targets using a nuclear acid stain. NK cells were purified from thawed PBMCs by negative selection following manufacturer’s instructions (StemCell, 17995) with an average NK cell purity rate of over 80% across all samples. K562 cells (Merck) were cultured at a concentration of 1 × 10^5^–2 × 10^5^ cells/mL in a humidified 5% CO_2_ incubator at 37 °C. On the day of the experiment, K562 cells were labelled with CFSE (0.5uM) for 10 min at 37 °C and plated in 96-well plate at 10,000 cells per well. Varying dilutions of NK cells were added for an effector:target (E:T) ratio of 5:1, 2.5:1, 1.25:1, and 0.625:1. Controls for each experiment included: target cells only to account for spontaneous target death, effectors only to monitor spontaneous effector death, and targets treated with 0.1% Tween to quantify maximum lysis. The effectors and targets were co-incubated for 4 h in a humidified 5% CO2 incubator at 37 °C. At the end of the incubation DRAQ7 dye (3uM final concentration) was added to each well for 10 min at 37 °C, followed by acquisition of flow cytometric data. The percentage of dead targets was calculated as [(percent experimental lysis − percent spontaneous lysis)/(percent maximum lysis − percent spontaneous lysis)] × 100. Samples with > 40% spontaneous effector death were excluded from analysis.

### Apoptosis assay

Thawed PBMCs (0.5 × 10^6^ cells/well) from JSLE patients and controls were incubated in complete media or in complete media containing 1000 IU/ml of IFN-α2b^36^ for 48 h^49^ prior to surface staining with antibodies BUV395-CD3 and PE-Cy7-CD56, for 20 min. To detect apoptosis using the FITC Annexin V Apoptosis Detection Kit (BD, 556547), cells were washed in binding buffer and stained with Annexin V and propidium iodide (PI) for 15 min as per manufacturer’s protocol. Acquisition of flow cytometric data on unfixed cells was performed within 1 h of staining.

### Statistical analysis of flow cytometric data

All data were analyzed using R software version 4.2.2 and plots were produced using the ggplot2 package^50^. Population distributions were visualized using density plots and qq-plots. Formal Shapiro–Wilk normality testing was also performed to assess normality. Two-sided Mann–Whitney U tests or Student’s t-tests were applied to test differences between two groups as appropriate depending on the data distribution. When comparing more than two groups Kruskall Wallis test with Dunn’s test post-hoc testing or two-way ANOVA with Tukey’s test were applied as appropriate. To correlate clinical and demographic parameters Spearman or Pearson correlation was used in accordance with the distribution of the data. Comparisons of sex, age, and ethnicity across the cohorts included in each immunophenotyping analysis are shown in Supplementary Table S7.

### Cell sorting and RNA isolation

Thawed PBMCs from 6 healthy controls, 7 patients with low activity JSLE (SLEDAI ≤ 4) and 5 patients with active JSLE (SLEDAI > 4) were stained with BUV395-CD4, APC-CD3, APC-H7-Live dead, BV785-CD8a, AF488-CD19, PE-CD56, PE-Cy7-CD14 antibodies as previously described and NK cells were isolated by FACS. RNA was extracted from NK cells using a PicoPure RNA isolation kit according to manufacturer’s instructions.

### RNA sequencing

RNA quality control and sequencing was performed by UCL Genomics. RNA integrity was confirmed using Agilent’s 4200 Tapestation. Samples were processed using the NEBNext® Single Cell/Low Input RNA Library Prep Kit for Illumina (New England Biolabs, E6420). cDNA libraries were generated using the SMART (Switching Mechanism at 5' End of RNA Template) technology with 10 PCR cycles. cDNA was then enzymatically sheared, end-repaired and A-tailed before ligation of an indexed adaptor containing a Unique Molecular Identifier (IDT). Dual ligated fragments were enriched with a limited-cycle PCR (8 cycles) and R5 Primers (Swift BioScience). Samples were sequenced on a NextSeq 2000 P2 100 cycle flow-cell (Illumina) using a 101 bp single end with an 8 bp sample index read and an 8 bp UMI read. Run data were demultiplexed and converted to fastq files using Illumina’s bcl2fastq Conversion Software v2.20.

Transcript abundance was estimated using a custom Galaxy pipeline developed by UCL Genomics. Sequencing read adapters were trimmed and sequencing data quality control was performed using fastp before being aligned to the human genome (UCSC hg38) with RNA-STAR (2.5.2b). Aligned reads were then UMI deduplicated using JE-Suite (1.2.1). Reads per transcript were estimated using FeatureCounts by counting uniquely mapped reads that fall within coding or UTR regions of the genome. All sequence and annotation data were obtained from the Illumina iGenomes repository.

### Transcriptional data analysis

Statistical analysis and visualisation of transcriptional data was performed using R software and Bioconductor packages^51^ including DESeq2^52,53^ and ComplexHeatmap^54^. Normalization and differential analysis were conducted according to the DESeq2 model and package, controlling for sex and age in all comparisons. The p-values obtained were corrected for multiple testing using the Benjamini and Hochberg method. An adjusted p-value of less than 0.05 was used to identify differentially expressed genes (DEGs).

### Pathway and gene set enrichment analysis

Pathway enrichment analysis was performed using the Metascape gene annotation and analysis resource^55^ and GO biological process ontology catalogue. Upregulated DEG and downregulated DEG gene lists were analysed separately with significance determined by a p-value cut-off of 0.01 and a minimum enrichment score of 1.5. As a complementary approach, Gene Set Enrichment Analysis (GSEA) of all genes analysed based on differential expression rank was performed using GSEA 4.2.2 software^56^ with the hallmark MSigDB gene set collection^57^. Statistical significance of the enrichment scores (ES) was determined by gene set permutation. Gene set enrichment was deemed significant at false discovery rate (FDR) q value < 0.25.

### Supplementary Information

Supplementary Information.

## Data Availability

The immunophenotyping data used in this study can be made available from the corresponding author upon reasonable request. RNA sequencing data can be found at ArrayExpress repository (accession number: E-MTAB-13422). This data will be available from manuscript publication date.
