# Supplementary material for: Active juvenile systemic lupus erythematosus is associated with distinct NK cell transcriptional and phenotypic alterations
Source: Sci Rep. 2024 Jun 6;14:13074. doi: 10.1038/s41598-024-62325-3 (PMC11156641; doi:10.1038/s41598-024-62325-3)
Supplement: Supplementary file 1 — Supplementary Information. [file 41598_2024_62325_MOESM1_ESM.pdf]

Active juvenile systemic lupus erythematosus is associated with distinct NK cell transcriptional and phenotypic alterations

Anna Radziszewska[1,2*], Hannah Peckham[1,2], Nina M de Gruijter[1,2], Restuadi Restuadi[1], Wing Han Wu[1,3], Elizabeth C Jury[2], Elizabeth C Rosser[1,2†*], Coziana Ciurtin[1,2†*]

Supplementary Information

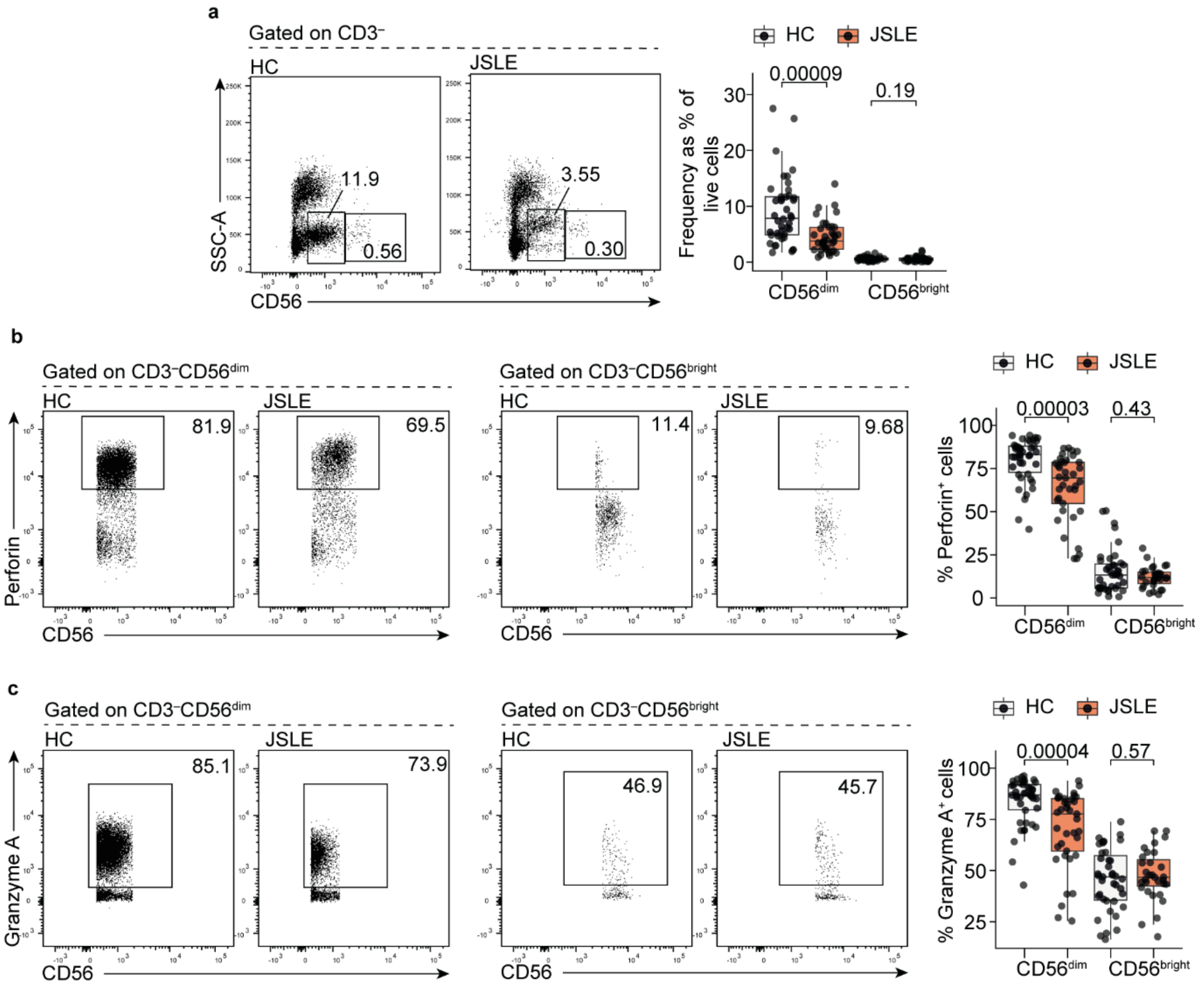

Supplementary Figure S1. CD56^{dim} cells and CD56^{dim} cells expressing perforin and granzyme A are reduced in JSLE. Representative flow plots and boxplots showing **(a)** CD56^{dim} and CD56^{bright} subpopulation frequencies in healthy controls (n=42) and patients with JSLE (n=37), **(b)** frequencies of perforin and **(c)** granzyme A in CD56^{dim} (HC n=42, JSLE n=37) and CD56^{bright} cells in healthy subjects (n=39) and patients with JSLE (n=31). Box plots shown median ± IQR. p-values calculated using Mann-Whitney U test.

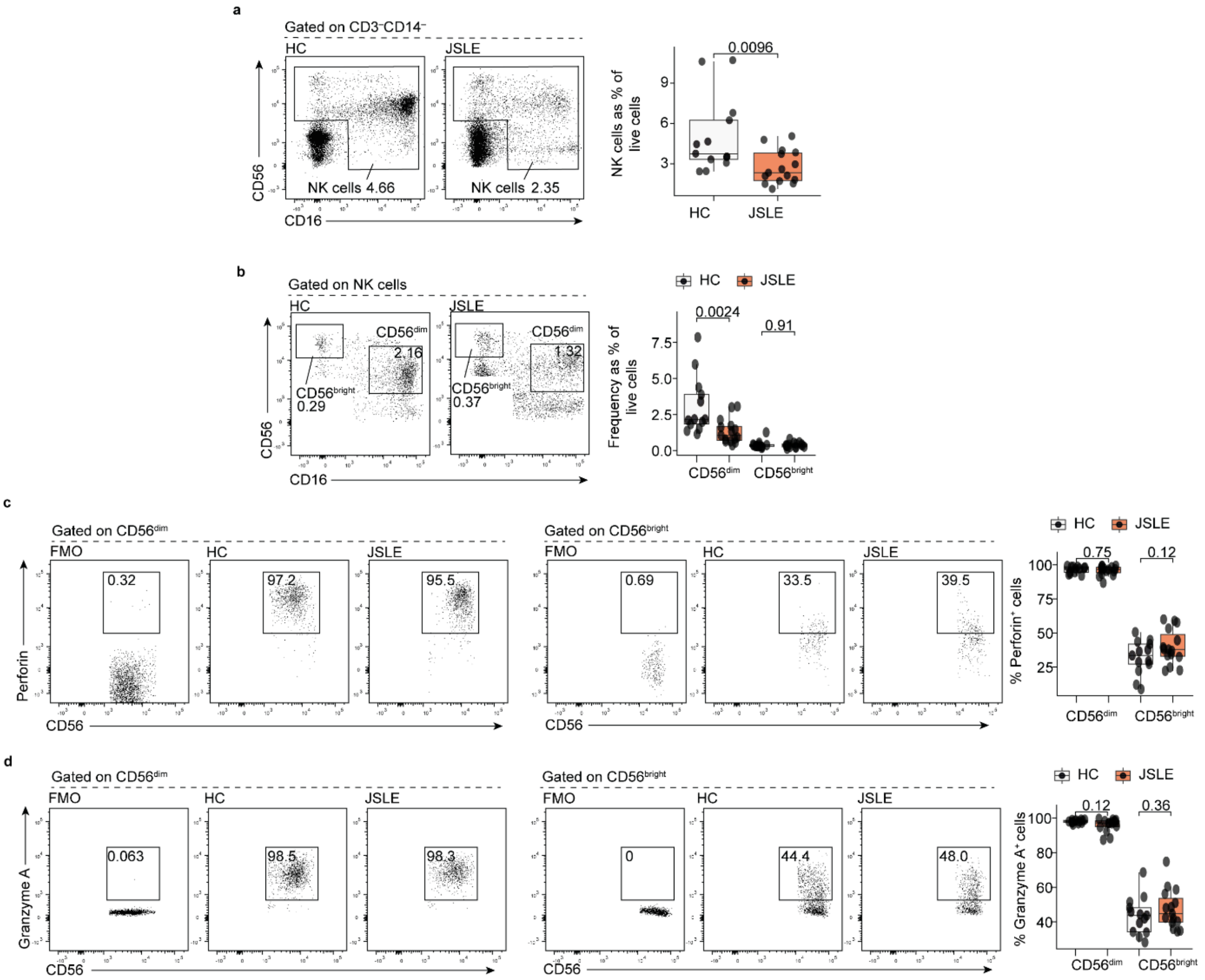

Supplementary Figure S2. CD16^{+}CD56^{dim} cells are reduced in JSLE. Representative flow plots and boxplots showing **(a)** total NK cells (expressed as frequency of live CD3^{-}CD14^{-} cells), **(b)** CD16^{+}CD56^{dim} and CD16^{-}CD56^{bright} subpopulations **(c)** perforin and **(d)** granzyme A in CD56^{dim} and CD56^{bright} cells in healthy subjects (n=13) and JSLE patients (n=15). Box plots shown median ± IQR. p-values calculated using Mann-Whitney U test or t-test as appropriate.

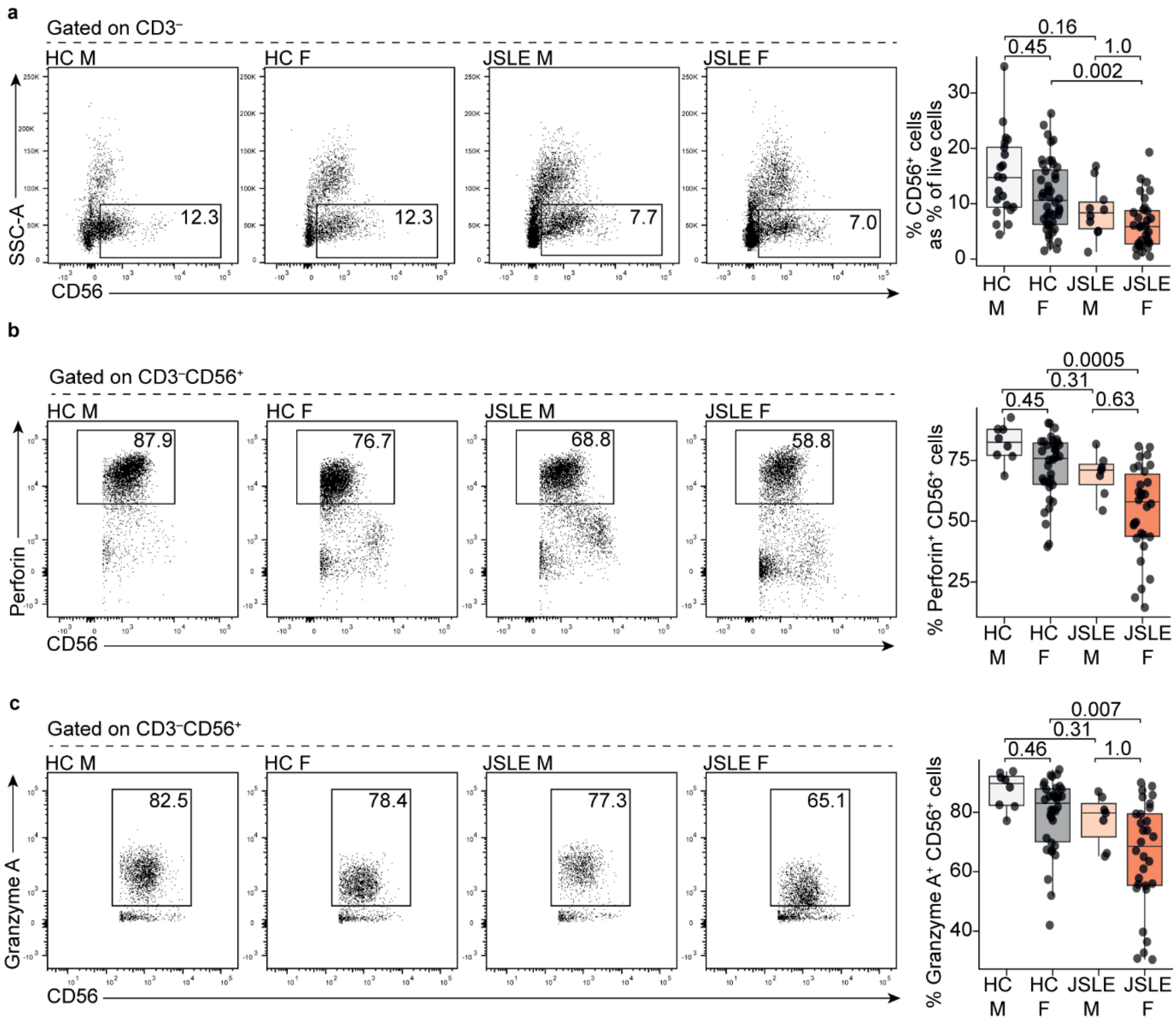

Supplementary Figure S3. No sex differences in frequencies of total CD56⁺ NK cells, perforin, or granzyme A expressing CD56⁺ NK cells in JSLE and healthy controls. Representative flow diagrams and boxplots quantifying frequencies of **(a)** CD56⁺ NK cells expressed as a percentage of all live cells (HC M: n=21, HC F: n=44, JSLE M: n=10, JSLE F: n=32), CD56⁺ NK cells expressing **(b)** perforin (HC M: n=8, HC F: n=34, JSLE M: n=7, JSLE F: n=30) and **(c)** granzyme A (HC M: n=8, HC F: n=34, JSLE M: n=7, JSLE F: n=30), across sex M= male, F= female. Boxplots show median ± IQR. p-values calculated using Dunn's test with Bonferroni correction.

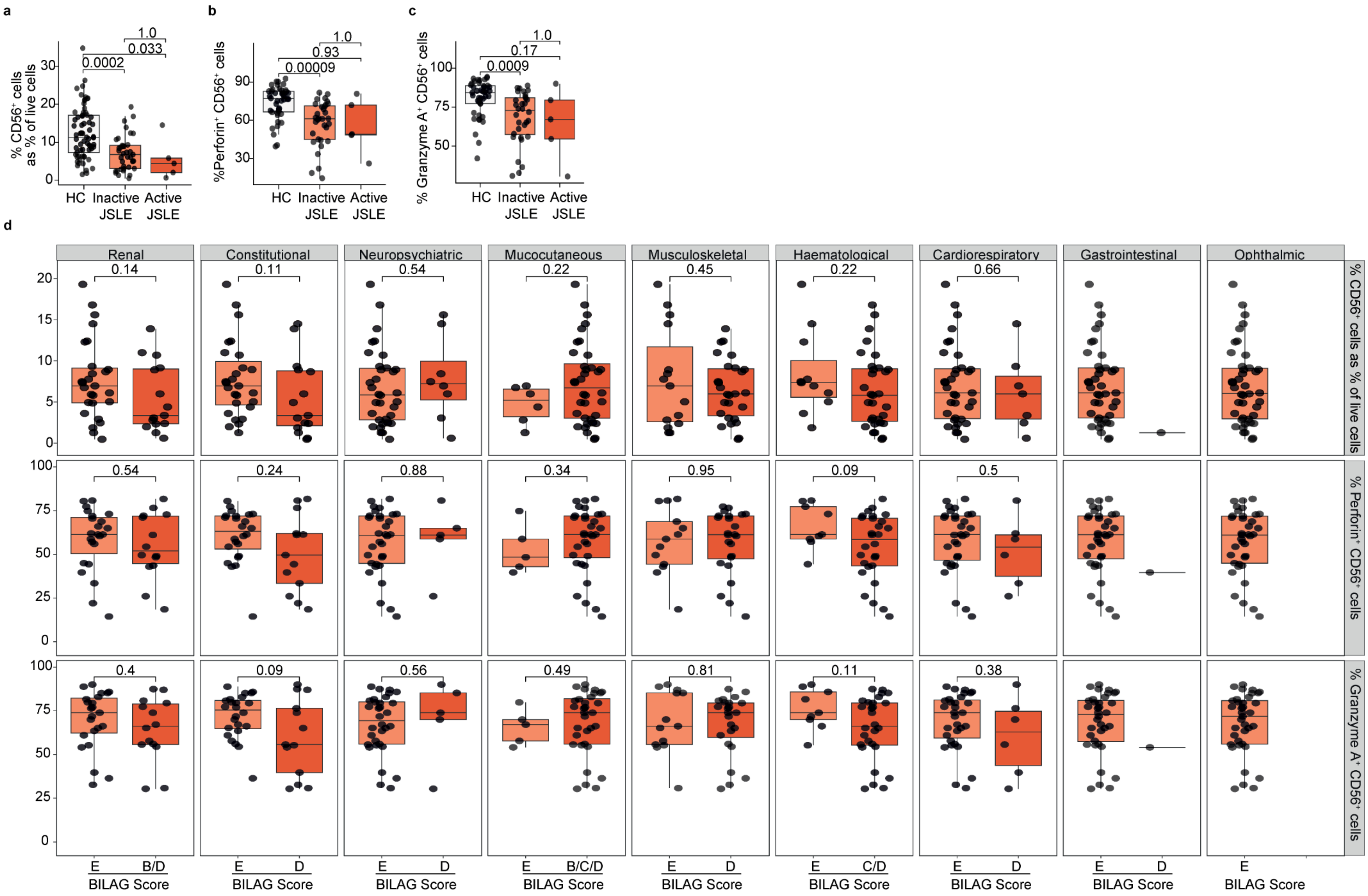

Supplementary Figure S4. Reductions in CD56⁺ NK cell populations are not associated with JSLE BILAG clinical disease activity. Boxplots showing frequencies of **(a)** total NK cells expressed as a percentage of all live cells (HC n=65, Inactive JSLE n= 37, Active JSLE n=5). **(b)** Perforin⁺ NK cells and **(c)** Granzyme A⁺ NK cells stratified based on BILAG score (HC n=42, Inactive JSLE n= 32, Active JSLE n=5). Active disease was defined as at least one BILAG B score (Global BILAG score ≥8) in any of the organ domains. **(d)** Boxplots showing frequencies of total NK cells expressed as percentage of live cells, perforin⁺ NK cells, and granzyme A⁺ NK cells in JSLE patients stratified into those with current or previous organ involvement (BILAG scores A-D) and those who never experienced disease involvement in the specified organ domain (BILAG score E) across the 9 organ systems comprising the BILAG score. All boxplots show median ± IQR and p values calculated using (a-c) Dunn's multiple comparison test or ANOVA with Tukey test or (d) two-sided Student's t-test, Welch test or Mann-Whitney test, as appropriate.

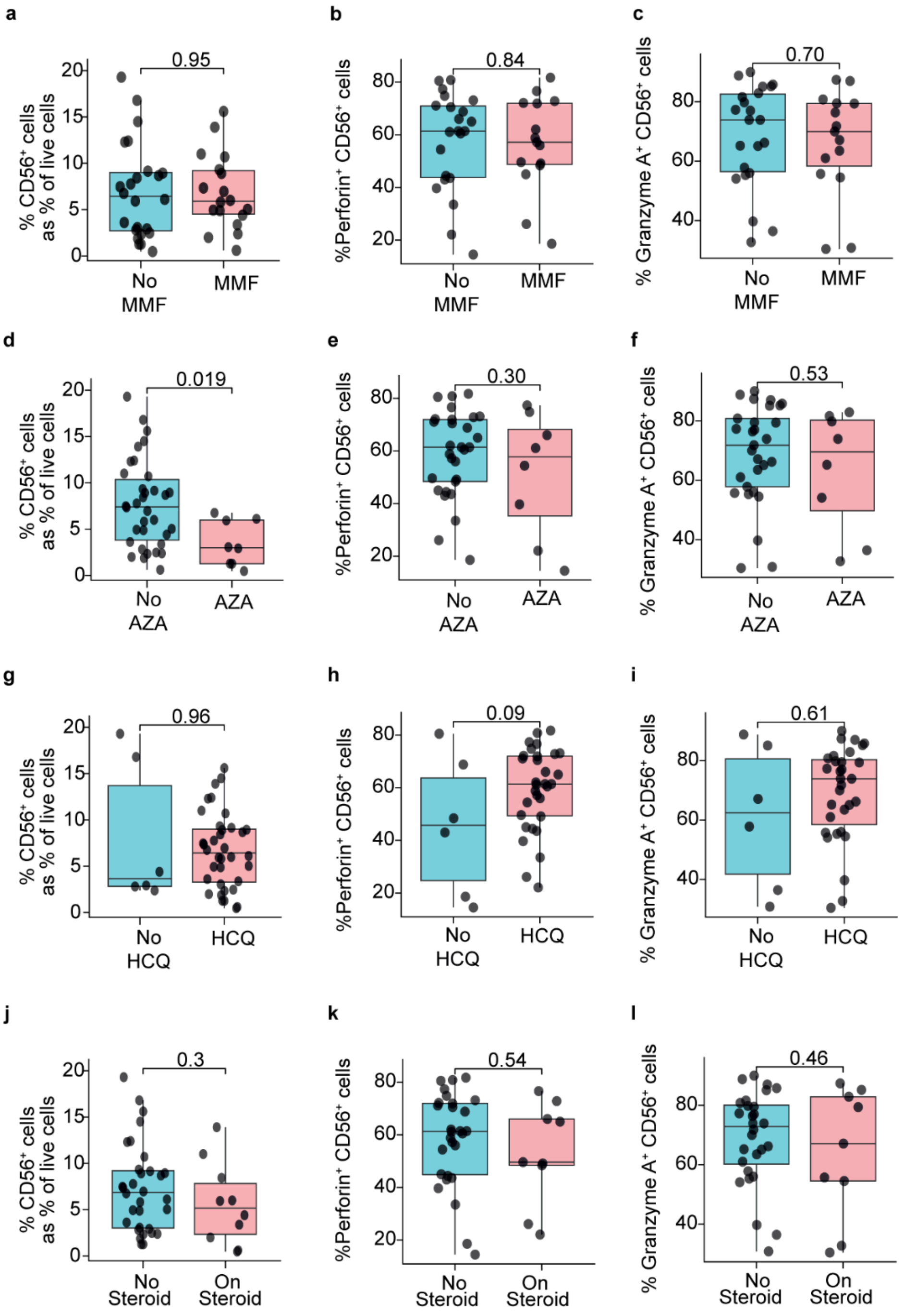

Supplementary Figure S5. Reduction in NK cells in JSLE is associated with azathioprine treatment. Boxplots showing frequencies of total CD56^{+} NK expressed as a percentage of all live cells and frequencies of NK cells expressing perforin and granzyme A in JSLE patients stratified on treatment with **(a, b, c)** mycophenolate mofetil (MMF) (NK: no MMF n=24, MMF n=18, NK perforin/granzyme A: no MMF n=22, MMF n=15), **(d, e, f)** azathioprine (AZA) (NK: no AZA n=34, AZA n=8, CD56^{+} perforin/granzyme A (no AZA n=29, AZA n=8), **(g, h, i)** hydroxychloroquine (HCQ) (NK: no HCQ n=6, HCQ n=36, NK perforin/granzyme A: no HCQ n=6, HCQ n=21) and **(j, k , l)** oral prednisolone use at time of sampling (NK: no steroid n=32, steroid n=10, NK perforin/granzyme A: no steroid n=28, steroid n=9). All boxplots show median ± IQR and p-values were calculated using two-sided Student’s t-test or Mann-Whitney test as appropriate.

Supplementary Table S1. Clinical characteristics of JSLE patients included in RNA-Seq analysis.

Sample Code	Diagnosis	Sex	Age at Sampling	Age Onset	Disease Duration	SLEDAI	BILAG General	Activity SLEDAI	Activity BILAG	BILAG Comment	SLICC	PGA (/3)	C3 Result	C4 Result	dsDNA Result Old	dsDNA Result New	dsDNA Result Interpretation	ESR	Leukocyte Count	Lymphocyte Count	HCQ	MMF	MTX	Prednisolone	AZA	CYCLO	Prednisolone Dose mg	Rituximab Prior to Sample Date	Years since last rituximab	ANA Result	LA Result	Anti-CL IgM	Anti-CL IgG	ENA Result	ENA Type
JSLE101(3)	JSLE/APS	F	19.6	15.8	3.8	2	8	Inactive	Active	mucocutaneous B, constitutional D, neuropsychiatric D, cardiorespiratory D	0	1	1.42	NA	31	NA	Negative	25	5.93	1.32	YES	NO	NO	NO	NO	NO	NA	TRUE	3.8	Positive	Positive	12.8	1578	Negative	NA
JSLE112(4)	JSLE	F	18.4	11.2	7.2	0	0	Inactive	Inactive	mucocutaneous D, musculoskeletal D	NA	NA	NA	NA	29	NA	Negative	9	8.14	3.21	YES	NO	NO	NO	NO	NO	NA	FALSE	NA	Positive	Positive	1.5	5.2	Negative	NA
JSLE136(1)	JSLE	F	18.8	2.0	16.8	0	0	Inactive	Inactive	constitutional E, mucocutaneous D, neuropsychiatric E,musculoskeletal D,cardiorespiratory E, gastrointestinal E, ophthalmic E, renal E, haematological E	0	0	1.19	0.25	10	NA	Negative	2	6.89	1.89	YES	NO	NO	NO	YES	NO	NA	FALSE	NA	Positive	Negative	1.0	2.6	Negative	NA
JSLE30(7)	JSLE	M	26.2	8.0	18.2	2	0	Inactive	Inactive	constitutional D, mucocutaneous D, neuropsychiatric E,musculoskeletal E, cardiorespiratory E, gastrointestinal E, ophthalmic E, renal D, haematological D	NA	NA	0.77	0.22	25	NA	Negative	5	3.26	0.64	YES	YES	NO	NO	NO	NO	NA	FALSE	NA	NA	Negative	4.9	5.4	NA	NA
JSLE65(1)	JSLE/APS	M	21.7	14.8	6.9	0	0	Inactive	Inactive	constitutional E , mucocutaneous D, neuropsychiatric E,musculoskeletal D,cardiorespiratory E, gastrointestinal E, ophthalmic E , renal D, haematological D	NA	NA	1.23	NA	26	NA	Negative	5	NA	2.53	YES	YES	NO	NO	NO	NO	NA	FALSE	NA	Negative	Positive	0.7	14.8	Negative	NA
JSLE84(6)	JSLE	F	21.98	8.7	13.3	0	0	Inactive	Inactive	mucocutaneous D, musculoskeletal D	0	0	1.45	0.33	NA	NA	Not Assessed	21	5.98	1.69	YES	YES	NO	NO	NO	NO	NA	FALSE	NA	NA	NA	NA	NA	NA	NA
JSLE86(4)	JSLE	F	21.8	13.7	8.1	0	0	Inactive	Inactive	constitutional D, mucocutaneous D, neuropsychiatric E,musculoskeletal D,cardiorespiratory D, gastrointestinal E, ophthalmic E, renal E, haematological D	0	0	1.16	0.26	NA	6	Negative	5	4.71	1.73	YES	YES	NO	NO	NO	NO	NA	FALSE	NA	NA	NA	NA	NA	NA	NA
JSLE18(11)	JSLE	F	25.9	18.0	7.9	8	8	Active	Active	renal B, haematological D, musculoskeletal D	NA	NA	0.64	0.19	145	NA	Positive	12	NA	0.73	NO	YES	NO	YES	NO	YES	7.50	TRUE	4.9	NA	Negative	3.6	1.8	NA	NA
JSLE47(13)	JSLE	F	28.2	12.4	15.8	6	8	Active	Active	constitutional E, mucocutaneous B , neuropsychiatric E,musculoskeletal D,cardiorespiratory E, gastrointestinal E, ophthalmic E, renal E, haematological D	0	0	NA	NA	162	NA	Positive	2	3.38	1.76	YES	YES	NO	NO	NO	NO	NA	FALSE	NA	NA	NA	NA	NA	Negative	NA
JSLE63(3)	JSLE	F	18.2	10.2	7.9	6	9	Active	Active	renal B, mucocutaneous C, musculoskeletal D, haematological D	NA	NA	0.45	NA	195	NA	Positive	35		1.2	YES	YES	NO	YES	NO	NO	10	FALSE	NA	Positive	Negative	0.5	2.2	Positive	anti-Ro
JSLE76(1)	JSLE/JDM	M	18.2	18.0	0.2	10	18	Active	Active	constitutional C, mucocutaneous B, musculoskeletal B, haematological C	NA	NA	0.33	0.32	2827	NA	Positive	38	NA	0.68	NO	NO	NO	NO	NO	NO	NA	FALSE	NA	Positive	Negative	3.0	11.2	Positive	anti-RNP, anti-Ro, anti-Sm
JSLE92(1)	JSLE	M	15.39	15.3	0.1	8	24	Active	Active	mucocutaneous B, haematological B, constitutional B	NA	NA	0.62	0.05	6907	NA	Positive	44	7.78	0.7	YES	NO	NO	YES	NO	NO	30	FALSE	NA	Positive	Negative	10.6	8.9	Negative	NA

Data Key

Column Name	Description
**Sample Code**	Lab code assigned to individual patient
**Diagnosis**	Patient diagnosis
**Sex**	Patient biological sex
**Age at Sampling**	Age at time sample was taken in years
**Age Onset**	Age at onset of disease (in years)
**Disease Duration**	Duration of disease in years
**SLEDAI**	SLEDAI-2K score (Systemic Lupus Erythematosus Disease Activity Index, range 0-105)
**BILAG General**	Global BILAG (British Isles Lupus Assessment Group) score, range 0-108
**ActivitySLEDAI**	Disease activity interpretation as assessed by SLEDAI, Active SLEDAI > 4, Inactive SLEDAI <= 4
**ActivityBILAG**	Disease activity interpretation as assessed by Global BILAG score, Inactive = BILAG 0, Active = BILAG 8+
**BILAG Comment**	BILAG score broken down for each organ system
**SLICC**	Systemic Lupus International Collaborating Clinics/American College of Rheumatology (SLICC/ACR) Damage Index (SDI) (on a scale of 0-47)
**PGA**	Physician Global Assesment (for JSLE only, on a scale of 0-3)
**C3 Result**	Complement 3 lab test result (normal range 0.9-1.8g/L)
**C4 Result**	Complement 4 lab test result (normal range 0.1-0.4g/L)
**dsDNA Result Old**	Double stranded DNA antibody titres (normal range 0-50 IU/mL). Old antibody test performed until end of March 2021, then it was switched completely to the new lab test.
**dsDNA Result New**	Double stranded DNA antibody titres IgG only (normal range 0-10 IU/mL). New dsDNA antibody test introduced in summer of 2020, performed concurrently with old test until end of March 2021, used exclusively from then onwards.
**dsDNA Result Interpretation**	Interpretation of dsDNA result based on range of whichever test was available at the time. If no concordance between new and old test, interpretation was made using new test result

Column Name	Description
**ESR**	Erythrocyte sedimentation rate (normal range 1-7 mm/hr)
**Leukocyte Count**	Leukocyte count (normal range 3-10x10^9/L)
**Lymphocyte Count**	Blood lymphocyte count (normal range 1.2-3.65x10^9/L)
**HCQ**	Is the patient currently on hydroxychloroquine?
**MMF**	Is the patient currently on mycophenolate mofetil?
**MTX**	Is the patient currently on methotrexate?
**Prednisolone**	Is the patient currently on prednisolone?
**AZA**	Is the patient currently on azathioprine?
**CYCLO**	Is the patient currently on cyclophosphamide?
**Prednisolone Dose mg**	Prednisolone dose in mg if patient is on prednisolone
**Rituximab Prior to Sample Date**	Has the patient been on B cell depletion therapy prior to date of sample? Patients on B cell depletion within a year pre sample date have been excluded.
**Years since last rituximab**	Number of years since last B cell depletion therapy
**ANA Result**	Anti nuclear antibody test result performed +/- 1 year of sample date. If no ANA test performed within this time frame, it is reported as NA.
**LA Result**	Lupus anticoagulant test result result performed +/- 1 year of sample date. If no LA test performed within this time frame, it is reported as NA.
**Anti-CL IgM**	Anti-cardiolipin IgM antibody test result (normal range 0-12 MPLU) performed +/- 1 year of sample date.
**Anti-CL IgG**	Anti-cardiolipin IgG antibody test result (normal range 0-12 GPLU) performed +/- 1 year of sample date.
**ENA Result**	Extractible nuclear antigen test result performed +/- 1 year of sample date. If no ENA test performed within this time frame, it is reported as NA.
**ENA Type**	If ENA is positive, type of ENA detected

Supplementary Table S2. Demographic characteristics of healthy subjects and JSLE patients in NK cell transcriptional analysis.

	**HC** Number (% / range)	**Inactive JSLE** Number (% / range)	**Active JSLE** Number (% / range)	**p-value**
Total number	6	7	5	-
Female:Male	4:2	5:2	3:2	1.0
Median age (years)	22.0 (16.1-24)	21.7 (18.4-26.2)	18.2 (15.4-28.2)	1.0
**Ethnicity (%)**				
White	2 (33%)	3 (43%)	1 (20%)	0.83
Black	0 (0%)	1 (14%)	1 (20%)	0.73
South Asian	2 (33%)	2 (29%)	1 (20%)	1.0
East Asian	2 (33%)	1 (14%)	0 (0%)	0.59
Other	0 (0%)	0 (0%)	2 (40%)	0.07

p-values calculated using Fisher's exact test (sex and ethnicity) or one-way ANOVA (age).

Supplementary Table S3. Comparison of demographic and clinical characteristics between NK immunophenotyping and transcriptional analysis cohorts.

	Phenotyping Cohort	RNAseq Cohort	p-value
**Sex ratio F:M**			
HC	45:21	4:2	1.0
Inactive JSLE	29:10	5:2	1.0
Active JSLE	4:0	3:2	0.44
**Median age in years (range)**			
HC	19.5 (15.2-32.2)	22.0 (16.1-24)	0.70
Inactive JSLE	21.4 (15.6-29.8)	21.7 (18.4-26.2)	0.78
Active JSLE	26.4 (18.2-28.2)	18.2 (15.4-28.2)	0.33
**Average SLEDAI (range)**			
Inactive JSLE	1.0 (0-4)	0.6 (0-2)	0.60
Active JSLE	7.5 (6-10)	7.6 (6-10)	0.94
**Average BILAG (range)**			
Inactive JSLE	0.1 (0-1)	0.1 (0-1)	0.43
Active JSLE	8.4 (8-9)	10.6 (8-24)	0.55
**AZA treatment Yes:No (%Yes)**			
Inactive JSLE	8:31 (19%)	1:6 (8%)	1.0
Active JSLE	0:4 (0%)	0:5 (0%)	-

p-values calculated using Fisher's exact test (sex and AZA treatment), unpaired Mann-Whitney U test (age: HC, SLEDAI: inactive JSLE, BILAG) or unpaired Student's t-test (age: inactive and active JSLE, SLEDAI: active JSLE) in accordance with the distribution of the data.

Table S4. Top 10 upregulated and downregulated genes in active JSLE patients compared to healthy controls.

Gene Name	Gene Function	Adjusted p-value	Log2 Fold Change
Upregulated (FDR adjusted p-value <0.05, FC>0)			
OAS1	Interferon-induced antiviral response	7.73E-12	2.71
LGALS3BP	Integrin-mediated cell adhesion	4.86E-09	4.00
PLSCR1	Phospholipid movement and distribution	9.97E-08	2.00
SPATS2L	RNA binding	1.08E-07	2.57
XAF1	Negative regulation of apoptosis inhibition	3.18E-06	1.45
OAS3	Interferon-induced antiviral response	3.80E-06	2.21
NIPSNAP3A	Vesicular transport	1.05E-05	1.14
CCR1	Chemokine receptor	1.58E-05	2.30
HLA-DRB1	Antigen presentation	3.73E-05	3.24
ANTXR2	Extracellular matrix adhesion	3.75E-05	1.64
Downregulated (FDR adjusted p-value <0.05, FC<0)			
PDZD4	Ubiquitin protein ligase activity (predicted)	9.60E-06	-1.53
SLC25A29	Mitochondrial transport	0.00011	-2.23
ATP9A	Negative regulation of exosomal secretion, endocytic recycling	0.00029	-1.67
VAMP2	Vesicle fusion and transport	0.00038	-0.63
NCKAP1	Actin filament reorganization	0.00081	-1.33
HKR1	Negative regulation of transcription (predicted)	0.0018	-0.86
TPRG1L	Glutamatergic synaptic transmission (predicted)	0.0019	-0.66
TMEM255A	Response to bacterium (predicted)	0.0019	-2.24
ZNF154	Regulation of transcription	0.0026	-1.49
NINL	Microtubule organization	0.0032	-2.58

Table S5. Differentially expressed genes in JSLE patients with low disease activity compared to healthy controls.

Gene Name	Gene Function	Adjusted p-value	Log2 Fold Change
Upregulated (FDR adjusted p-value <0.05, FC>0)			
LOC100507195	Unknown	0.00011	1.46
LGALS3BP	Modulation of cell-cell and cell-matrix interactions	0.00035	2.76
POLR2J2	Messenger RNA synthesis	0.0059	4.86
Downregulated (FDR adjusted p-value <0.05, FC<0)			
C16orf72 (HAPSTR1)	Cellular stress response	0.031	-0.66

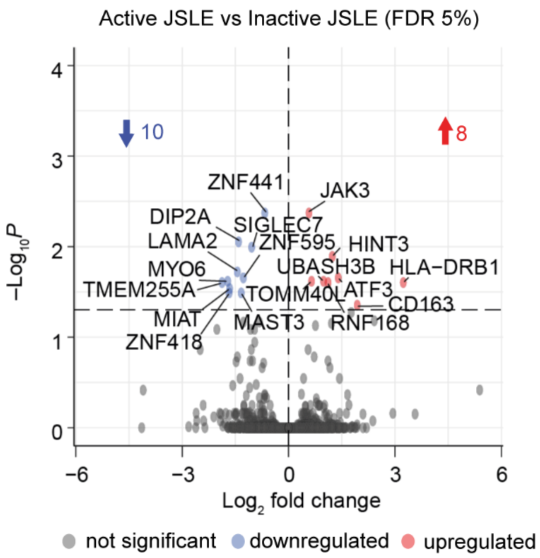

Supplementary Figure S6. NK cell transcriptional differences in active JSLE compared to JSLE patients with low disease activity. Volcano plot showing differences in gene expression from RNA sequencing in NK cells from active JSLE (n=5) vs inactive JSLE (n=7). Blue and red points represent statistically significant DEGs below the FDR adjusted p-value threshold of 0.05. Blue and red arrows indicate number of downregulated and upregulated genes, respectively.

Table S6. Differentially expressed genes in active JSLE patients compared to JSLE patients with low disease activity.

Gene Name	Gene Function	Adjusted p-value	Log2 Fold Change
Upregulated (FDR adjusted p-value <0.05, FC>0)			
JAK3	Tyrosine kinase, cytokine receptor-mediated intracellular signal transduction	0.0043	0.57
HINT3	Nucleotide hydrolase and transferase	0.013	1.23
ATF3	Transcription factor involved in cellular stress response	0.022	1.40
UBASH3B	Inhibition of epidermal growth factor receptor and platelet-derived growth factor receptor endocytosis	0.024	0.99
TOMM40L	Protein import into mitochondrial matrix	0.024	0.65
HLA-DRB1	MHC class II antigen presentation	0.025	3.23
RNF168	Ubiquitin ligase involved in DNA double-strand break repair	0.025	1.11
CD163	Haemoglobin scavenger receptor in monocytes (NK cell function unknown)	0.044	1.94
Downregulated (FDR adjusted p-value <0.05, FC<0)			
ZNF441	Regulation of transcription	0.0043	-0.67
DIP2A	Axon patterning in the central nervous system	0.0089	-1.41
SIGLEC7	Cell adhesion (predicted), enabling sialic acid binding activity (predicted)	0.010	-1.04
LAMA2	Mediation of attachment, migration and organization of cells into tissues during embryonic development	0.019	-1.44
ZNF595	Regulation of transcription	0.022	-1.28
MYO6	Intracellular vesicle and organelle transport	0.024	-1.72
TMEM255A	Response to bacterium	0.025	-1.88
MIAT	Long non-coding RNA that may constitute a component of the nuclear matrix	0.029	-1.66
MAST3	Enablement of protein serine/threonine kinase activity, cytoskeleton organization, intracellular signal transduction and peptidyl-serine phosphorylation (predicted)	0.032	-1.34
ZNF418	Regulation of transcription	0.032	-1.68

Supplementary Table S7. Comparison of sex, age and ethnicity differences across immunophenotyped cohorts.

			Ethnicity				
**Data set**	**Sex**	**Age**	**White**	**Black**	**South Asian**	**East Asian**	**Other**
**Figure 1a, 2a, S3a**	0.18	0.39	0.32	0.33	0.16	0.24	1.00
**Figure 1b, 1c, 1d, 2b, 2c, S3b, S3c**	0.70	1.00	0.18	0.09	0.11	0.32	1.00
**Figure 1e**	0.42	1.00	0.78	0.04*	0.52	0.27	0.72
**Figure 3a-d**	0.48	1.00	0.64	1.00	0.08	0.08	-
**Figure 3e-f**	0.89	1.00	1.00	0.56	0.44	0.22	-
**Figure 4a**	0.27	0.47	0.53	0.41	0.15	0.62	0.44
**Figure 4c**	0.41	0.90	0.30	0.13	0.13	0.71	0.56
**Figure 4e**	0.41	0.90	0.30	0.13	0.13	0.71	0.56
**Figure 6**	0.74	1.00	0.65	1.00	0.14	0.21	-
**Supplementary Figure S1a, S1b CD56dim, S1c CD56dim**	0.70	1.00	0.18	0.09	0.11	0.32	1.00
**Supplementary Figure S1b CD56bright, S1c CD56bright**	0.97	0.76	0.10	0.16	0.15	0.45	1.00
**Supplementary Figure S2a, S2b, S2c**	0.87	1.00	0.26	0.48	0.21	0.60	0.48
**Supplementary Figure S4a**	0.41	0.38	0.39	0.36	0.07	0.50	0.49
**Supplementary Figure S4b, S4c**	0.63	0.66	0.23	0.14	0.06	0.64	0.62

p-values calculated using unpaired Mann-Whitney test (HC vs JSLE: sex and age for all except Figure 4 and S4), Fisher's exact test (HC vs JSLE ethnicity), or ANOVA with Tukey test (HC vs inactive JSLE vs active JSLE: sex and age Figure 4 and S4) *$p<0.05$
